# Comparison of Fermentation Behaviors and Characteristics of Tomato Sour Soup between Natural Fermentation and Dominant Bacteria-Enhanced Fermentation

**DOI:** 10.3390/microorganisms10030640

**Published:** 2022-03-17

**Authors:** Juan Li, Xiaoyu Wang, Wenyan Wu, Jingzhu Jiang, Dandan Feng, Yuanyuan Shi, Ping Hu

**Affiliations:** 1 School of Liquor and Food Engineering, Guizhou University, Guiyang 550025, China; juanli0216@163.com (J.L.); wwy18275266629@163.com (W.W.); jiangjingzhukk@163.com (J.J.); danfeng17885906867@163.com (D.F.); 15085497396@163.com (Y.S.); 2 College of Life Science, Guizhou University, Guiyang 550025, China; gzuwangxiaoyu@163.com

**Keywords:** tomato sour soup, natural fermentation, dominant bacteria, enhanced fermentation

## Abstract

In this study, the correlations between microbial communities with physicochemical properties and volatile flavor compounds (VFCs) during the fermentation of traditional tomato sour soup (CTN) are explored. The results of high-throughput sequencing (HTS) of CTN showed that *Lacticaseibacillus* (28.67%), *Enterobacter* (12.37%), and *Providencia* (12.19%) were the dominant bacteria in the first round of fermentation, while *Lacticaseibacillus* (50.11%), *Enterobacter* (13.86%), and *Providencia* (8.61%) were the dominant bacteria in the second round of fermentation. Additionally, the dominant fungi genera of the first fermentation were *Pichia* (65.89%) and *Geotrichum* (30.56%), and the dominant fungi genera of the second fermentation were *Pichia* (73.68%), *Geotrichum* (13.99%), and *Brettanomyces* (5.15%). These results indicate that *Lacticaseibacillus* is one of the main dominant bacteria in CTN. Then, the dominant strain *Lacticaseibacillus casei* H1 isolated from CTN was used as a culture to ferment tomato sour soup to monitor dynamic changes in the physicochemical properties and VFCs during enhanced fermentation of tomato sour soup (TN). The physicochemical analysis showed that, compared with CTN, the TN group not only produced acid faster but also had an earlier peak of nitrite and a lower height. The results of the GC–IMS analysis showed that the ester and alcohol contents in the TN group were 1.26 times and 1.8 times that of the CTN group, respectively. Using an O2PLS-DA analysis, 11 bacterial genera and 18 fungal genera were identified as the functional core flora of the CTN group flavor production, further verifying the importance of dominant bacteria for the production of VFCs. This study proved that enhanced fermentation not only shortens the fermentation cycle of tomato sour soup, but also significantly improves its flavor quality, which has great value in the industrial production of tomato sour soup and in the development of a vegetable fermentation starter.

## 1. Introduction

**Figure 1:**
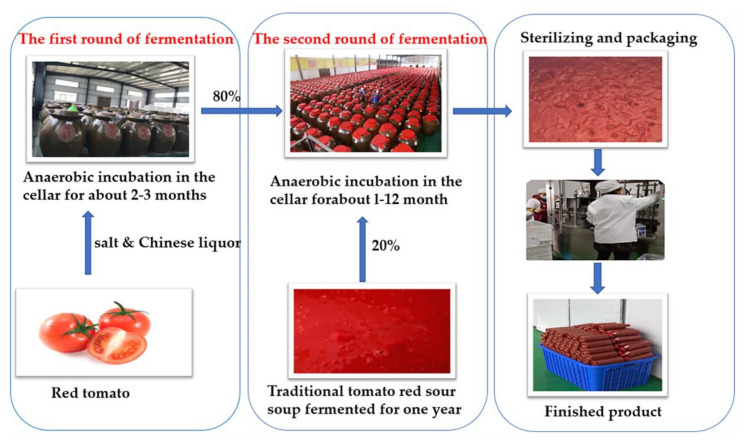
Production process of traditional fermented tomato sour soup.

Tomato sour soup is a traditional fermented vegetable sauce of the Dong and Miao Nationalities in China. Usually, traditional natural fermented tomato sour soup (CTN) is made from tomato and glutinous rice flour, baijiu, and other ingredients [1]. The fermentation process is divided into two rounds. The first round of fermentation is the natural fermentation of tomatoes into semi-finished products; in the second round of fermentation, traditional old sour soup is used as the starter and mixed with semi-finished products in a certain proportion. Figure 1 shows the production process of CTN. The first round of fermentation determines the acidity of CTN, which is an important stage of CTN taste and flavor formation [2]. Previous research showed that CTN is rich in a variety of organic acids [3], lycopene [4], phenols, minerals [5], and other nutrients, and has certain effects on the protection of non-alcoholic fatty liver disease [6], regulating intestinal flora, scavenging free radicals, and enhancing immunity [7]. At present, CTN is widely used as seasoning in processed dishes, such as sour soup fish, sour soup beef, hot pot seasoning, and other foods, highlighting the large market demand [8].

The formation of volatile flavor substances (VFCs) in tomato sour soup depends on the synergy between different microorganisms [9]. However, at present, the production of CTN is mainly natural fermentation, and the cycle ranges from 1 month to 12 months. Due to the absence of sterilization operations, the overgrowth of miscellaneous bacteria results in the accumulation of nitrite at the beginning of fermentation, resulting in the problem of quality instability between batches [10,11]. However, few studies focus on the succession of microbial flora during CTN fermentation, and the characterization of VFCs of CTN has not been thoroughly studied. The mechanism of the dominant flora on the formation of VFCs in CTN fermentation is unclear, resulting in inconsistent taste and flavor of different batches of products from the same manufacturer. The formation of VFCs in fermented foods is closely related to the microorganisms in the fermentation process. Therefore, selecting suitable microbial strains to standardize the fermentation process, shorten the fermentation cycle, and stabilize the flavor quality of the product is necessary. Although there have been studies on the flavor and microbial communities of sour soup, and the microbial flora [12,13,14,15,16], there are few studies on the VFCs of tomato sour soup that use the inoculation of dominant bacteria, or on the relationship between the change in microbial communities and VFC generation in the CTN fermentation process used to enhance fermentation. Therefore, improving the quality of CTN is necessary to study the succession of microbial communities, the change in VFCs, their relationship during CTN fermentation, and the influence of enhanced fermentation by dominant bacteria on the quality of tomato sour soup.

In recent years, high-throughput sequencing (HTS) and gas chromatography–ion mobility spectroscopy (GC–IMS) technologies have been widely used in the detection of food microorganisms and VFCs [17], such as in fermented vegetables [18], Ma Milk wine [19], natural fermented bean curd [20], etc. Recently, HTS was used to analyze bacteria in tomato sour soup, in which *Lacticaseibacillus* spp. was found to be the dominant bacteria [16]. The microbial structure of fermented foods often has an effect on flavor substances; however, the relationship between these microbiota and flavor is still poorly understood.

The aims of this study are to gain a more comprehensive understanding of the succession of microbial flora and the changes in the physicochemical indexes and VFCs during CTN fermentation; to reveal their interactions; and to clarify the effect of using a dominant strain to strengthen fermentation, thus improving the quality and safety of tomato sour soup and shortening the fermentation period, which has great value in the development of a fermentation starter and in the industrial production of tomato sour soup.

## 2. Materials and Methods

### 2.1. Preparation of the Starter

The dominant bacteria used in this research was *Lacticaseibacillus casei* H1 (CCTCC NO: M2016524), which was isolated and identified from samples of CTN [21]. The H1 strain was activated by MRS broth medium (Beijing Road and Bridge Technology Co., Ltd., Beijing, China), the cells were collected by centrifugation, and the concentration of the bacterial solution was adjusted to 10 ^9^ CFU/mL using 0.85% normal saline during use.

### 2.2. Sample Collection and Analysis

#### CTN Sample Collection

CTN was collected from a sour soup manufacturer factory in Qiandongnan, Guizhou province, China. The CTN samples were divided into two rounds of fermentation. The first round of fermentation included the following steps: place the freshly cleaned tomatoes into the fermentation tank, add salt, mix well, and ferment until the mixture becomes thick (2–3 months) [12]. The second round of fermentation included the following steps: beat the product obtained from the first round of fermentation (denoted as fermentation xd) and pour it into the fermentation tank; then, add 20% B (old sour soup, denoted as fermentation 0 d).

Samples were taken at Days 1 (C), 5 (D), 11 (E), and 19 (F) during the second round of fermentation. According to the method of Du et al. [22], the samples were prepared by mixing equal amounts of the mixture from five points (the surface, middle, and bottom of the four corners and the midpoint of the fermentation tank) and were collected in sterile sampling bottles, transported to the laboratory on dry ice, and stored at −80 °C without loss for further analysis.

The sample preparation scheme of the TN group is as follows: in the laboratory, a mixture of 93.55% beaten tomato, 2% prepared inoculated strain, 2% glutinous rice flour, 1.45% salt, and 1% liquor was encapsulated in a sterile tank and fermented naturally at room temperature (about 25 °C) [23] for 11 days; the samples (fermentation broth) were collected from sterile tanks on Days 0 (A), 1, 3 (B), 5 (C), 7 (D), 9, and 11 (E) and stored at −80 °C until analysis. All tomatoes were purchased from the local supermarket.

### 2.3. Extraction of Microbial Genome

The TGuide S96 magnetic bead method soil kit (Tiangen Biochemical Technology Co., Ltd., Beijing, China) (model: DP812) was used to extract DNA from CTN samples in six stages (A, B, C, D, E, and F). The quality of the extracted DNA was evaluated by spectrophotometer and 1.8% agarose electrophoresis (120 V, 40–45 min). The Monarch DNA Gel Recovery Kit was used to purify and recover the amplified products. The primers 27F_(16S-F): AGRGTTTGATYNTGG CTCAG and 1492R_(16S-R): TASGGHTACCTTGTTASGACTT were used to amplify the 16S full-length region of the bacteria. The primers ITS1F: CTTGGTCATTTAGAGGAAGTAA and ITS4: TCCTCGCTTATTGAT ATGC were used to amplify the full-length region of fungal ITS. The 16S full-length reaction system consists of a 30 μL mixture, which contains 1.5 μL of genomic DNA(8.5–17.6 ng/uL), 10.5 μL of NFW(Hydration Volume), 15 μL of KOD ONE MM(KOD One ^TM^ PCR Master Mix, Toyobo (Shanghai, China) Biotechnology Co., Ltd., Beijing, China), and 3 μL of the barcode primer pair. The ITS series full-length reaction system consists of a 30 μL mixture that contains 1.5 μL of genomic DNA(8.5–17.6 ng/uL), 11.7 μL of NFW, 15 μL of KOD ONE MM, and 1.8 μL of the barcode primer pair. The PCR parameters for the detection of the 16S full-length region primer samples were as follows: 95 °C pre-denaturation for 5 min; 30 cycles in total of 95 °C denaturation for 30 s, 55 °C annealing for 30 s, and 72 °C extension for 90 s; and 72 °C extension for 7 min. The PCR parameters for the detection of the primer samples in the full-length region of ITS were as follows: 95 °C pre-denaturation for 5 min; a total of 8 cycles of 95 °C for 1 min, 55 °C annealing for 30 s, and 72 °C extension for 45 s; 72 °C extension for 45 s; then a total of 24 cycles of 95 °C for 1 min, 60 °C denaturation for 30 s, and 72 °C annealing for 45 s; and 72 °C extension for 7 min.

### 2.4. Bioinformatics Analysis

Sequencing was based on the PacBio sequencing platform, using single-molecule real-time sequencing (SMRT Cell) to sequence the marker gene and then filter, cluster, or denoise the CCS (Circular Consensus Sequencing) sequences; to divide the OTUs/ASVs, and to perform species annotations. The abundance analysis reveals the species composition of the sample.

### 2.5. Analysis of the Physicochemical Indexes

For the pH values and total acid contents, the method of Wang Chan [24] was used with slight modifications. For the nitrite concentrations, the method of Jeffrey was used [25].

#### HPLC Detection of Organic Acids

For the determination of the organic acid content, the method of Tian Ya [26] was used with slight modifications. HPLC was performed on an Agilent Z0RBAX SB-AQ (4.6 mm × 250 mm, 5 μm, American Agilent Corporation (Santa Clara, CA, USA) column with an Agilent1260 VWD detector. In the mobile phase, according to the following parameters: KH_2_PO_4_ (0.02 mol/LKH_2_PO_4_ pH 2) the ratio of methanol was 95:5, the flow rate was 0.8 mL/min, the column temperature was 35 °C, the detection wavelength was 210 nm, and the sample volume was 10 uL. The experiment was conducted three times for each sample.

### 2.6. GC–IMS Analysis of VFCs

A FlavourSpec^®^ gas phase–ion mobility spectrometry (GC–IMS) combined instrument was used for testing. The GC–IMS instrument was equipped with a syringe and an autosampler unit for headspace analysis. Two grams of the tomato sour soup was placed in a 20 mL headspace injection bottle. The injection volume was set to 300 μL and the soup was incubated at 60 °C for 10 min. A quartz capillary column (MXT-5, 15 m × 0.53 mm × 1.00 μm) was used for chromatographic separation, the column temperature was maintained at 60 °C, and nitrogen was used as the carrier gas. The chromatographic separation was performed under isothermal conditions. The carrier gas flow rate started at 2 mL/min, lasting for 2 min, and then increased to 100 mL/min within 18 min. The total analysis time was 20 min.

### 2.7. Statistical Analysis

OriginPro 2019.9.1 software was used to draw a line chart of the physicochemical indexes of the tomato sour soup during the fermentation process. SPSS 26 software (IBM, Armonk, NY, USA) was used for data processing, Excel 2010 software was used for statistical tabulation of the data, SIMCA-14.1software (UMETRICS, Umeå, Sweden) was used for O2PLS-DA modeling to determine the relationship between microbial communities and VFCs, and Laboratory Analytical Viewer configured with GC–IMS equipment was used. The analysis software and the built-in NIST database and IMS database performed the qualitative analysis of tomato sour soup. All data were subjected to three replicate tests.

## 3. Results and Discussion

### 3.1. Abundances of Bacterial and Fungal Sequences in CTN

**Figure 2:**
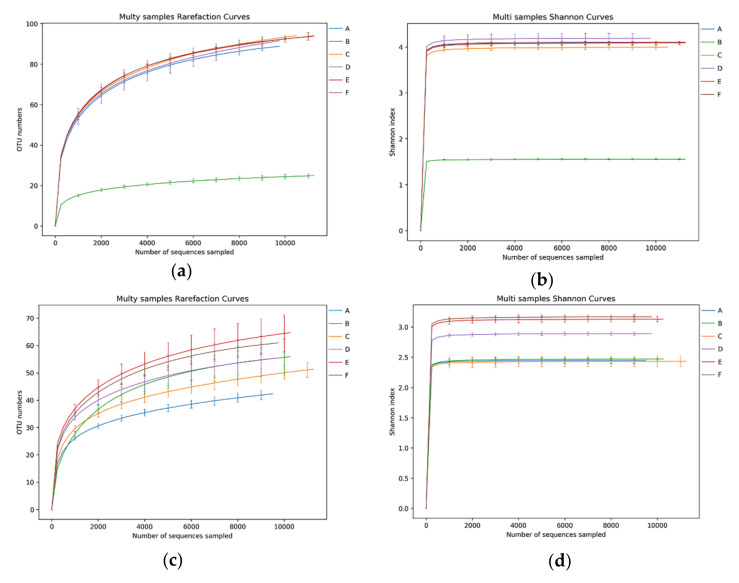
Rarefaction curve and Shannon curve of sequencing reads of (**a**,**b**) the bacterial 16S rDNA gene and (**c**,**d**) the fungal ITS rDNA gene from CTN samples.

HTS obtained a total of 234,420 16S rDNA and 211,920 ITS rDNA raw sequence reads from the six-stage CTN samples investigated. After quality-control processing such as splicing and filtering the original data, 207,092 high-quality bacterial labels and 208,315 high-quality fungal labels were obtained. Each sample covered an average of 12,878 valid bacterial labels and 10,185 valid fungal labels. The operating taxa (OTU) with 97% similarity reached the saturation platform (Figure 2a,c), and the coverage rate exceeded 99% (Table 1), indicating that most of the bacterial and fungal system types were identified. The sparse curve indicates that the sorting depth is sufficient, as shown in Figure 2a,c. The Shannon curve further shows that the number of sequences is sufficient and reasonable; Figure 2b,d contains most of the flora information in CTN samples [27]. As a sampling diversity estimator, the Shannon indices of bacteria and fungus are in the ranges of 1.55–4.19 and 2.43–3.17, respectively, and the Simpson indices of bacteria and fungus are in the ranges of 0.45–0.91 and 0.67–0.82, respectively, representing the approximate number of OTUs and their CTN. The uniformity of the distribution is provided in Table 1.

**Figure 3:**
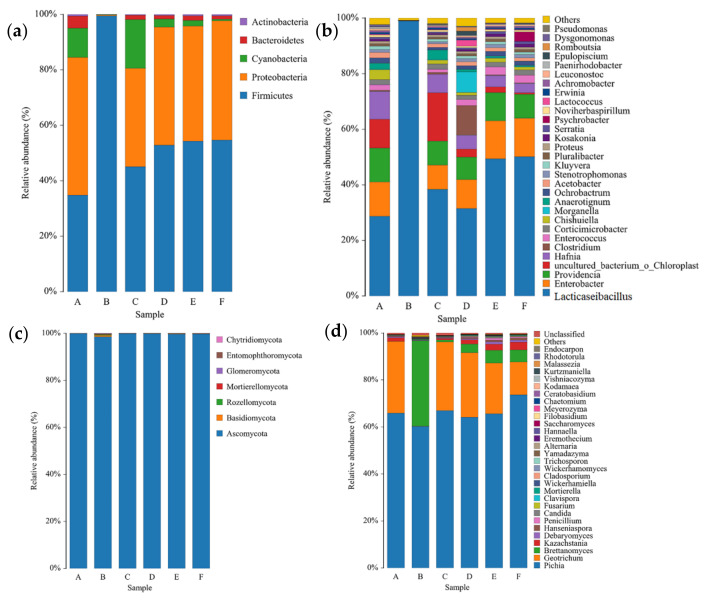
The relative abundance of bacteria and fungi in CTN at the phylum level (**a**,**c**) and genus level (**b**,**d**). Each phylum or genus is represented by a unique color. Each column represents a different studied sample of CTN (A–F). CTN refers to the traditional tomato sour soup sample.

The species richness indices (ACE index and Chao1 index) and diversity indices (Shannon index and Simpson index) are shown in Table 1, which shows that the community diversity of bacteria is generally richer than that of fungi (Table 1). The relative abundance analysis of bacteria and fungi shows the microbiota of CTN samples (Figure 3).

As shown in Figure 3a, a total of five bacterial phyla were detected in CTN at the bacterial phylum level, with *Firmicutes* (34.82–54.29%) and *Proteobacteria* (49.66–43.04%) being the major phyla found during the fermentation of CTN (Figure 3a); within a fermentation time of one year, *Firmicutes* accounted for 99.51% of the bacteria in old sour soup (B). These results are consistent with the results of *Firmicutes* and *Proteobacteria*, which are the most common dominant bacteria of fermented vegetables [28,29]. In addition, at the level of the fungal phylum (Figure 3c), seven phyla were detected during the fermentation of CTN. Among them, *Ascomycota* was the dominant phylum during fermentation, which is consistent with previous studies on fermented vegetables [30].

### 3.2. Bacterial Microbiota in CTN Samples

At the level of bacterial genera, the 276 species of bacteria in CTN were divided into 75 genera, of which 30 genera were detected (relative abundance > 0.1%). The dominant bacteria in the first round of fermentation (Figure 3b) are *Lacticaseibacillus* (28.67%, *Enterobacter* (12.37%), and *Providencia* (12.19%), and the dominant bacteria in the second round of fermentations are *Lacticaseibacillus* (50.11%), *Enterobacter* (13.86%), and *Providencia* (8.6%); in traditional old sour soup (B), *Lacticaseibacillus* accounted for 99.01% of the bacteria. The richness of *Lacticaseibacillus* (28.67–50.11%) gradually increased during the fermentation process, and the richness of *Enterobacter* was reduced (8.70%) after adding the samples of Stage B (one-year traditional old sour soup) to Stage A (12.37%) of the first round of fermentation and then increased to 13.86% in the second round of fermentation. The amount of *Providencia* fluctuated during the second round of fermentation, and the richness finally reached a maximum of 12.19% and a minimum of 8.6% in the A and D stages of the fermentation. With an extended fermentation time, the CTN species richness gradually decreased and *Lacticaseibacillus* dominated, playing an important role, which is consistent with the results of Wang Qiqi et al. [31]. These results indicate that *Lacticaseibacillus* produce a large amount of organic acid during the fermentation process, which not only gives CTN a soft sour taste and aroma, but also improves the nutritional value [28]. *Lacticaseibacillus* in particular produces lactic acid and a variety of antibacterial substances, such as bacteriocins, that inhibit the growth of pathogens and spoilage microorganisms during the fermentation process, increase the stability of the fermentation process, and improve the quality of CTN [32,33].

### 3.3. Fungal Microbiota in CTN Samples

At the level of fungal genera (Figure 3d), 193 species of fungi were divided into 51 genera during the fermentation process of CTN, of which 31 genera were detected (relative abundance > 0.1%). The dominant fungi in the first round of fermentation were *Pichia* (65.89%) and *Geotrichum* (30.56%). The dominant fungi in the second round of fermentation were *Pichia* (73.68%), *Geotrichum* (13.99%), and *Brettanomyces* (5.15%). The richness of *Pichia* and *Brettanomyces* gradually increased during the fermentation process, and the amount of *Geotrichum* showed a gradual decrease during the fermentation process.

In our study, *Geotrichum* was the predominant genus in all samples except for Sample B. *Brettanomyces* was the predominant genus in all samples except for Sample A. It can be seen that, during the second round of fermentation, *Geotrichum* was gradually replaced by *Brettanomyces*, and these yeasts may come directly from the rice wine added in the second round of fermentation. This finding indicates that the second round of fermentation of CTN in the fermenter co-ferments *Lacticaseibacillus* and yeasts, and that both *Lacticaseibacillus* and yeasts play key roles in the formation of the flavor compounds [12]. Current studies have shown that the synergistic fermentation of yeasts can reduce volatile acidity and higher aroma compounds, thereby improving the flavor and quality of wine [34].

### 3.4. Physicochemical Indices Analysis of TN Fermentation Process

**Figure 4:**
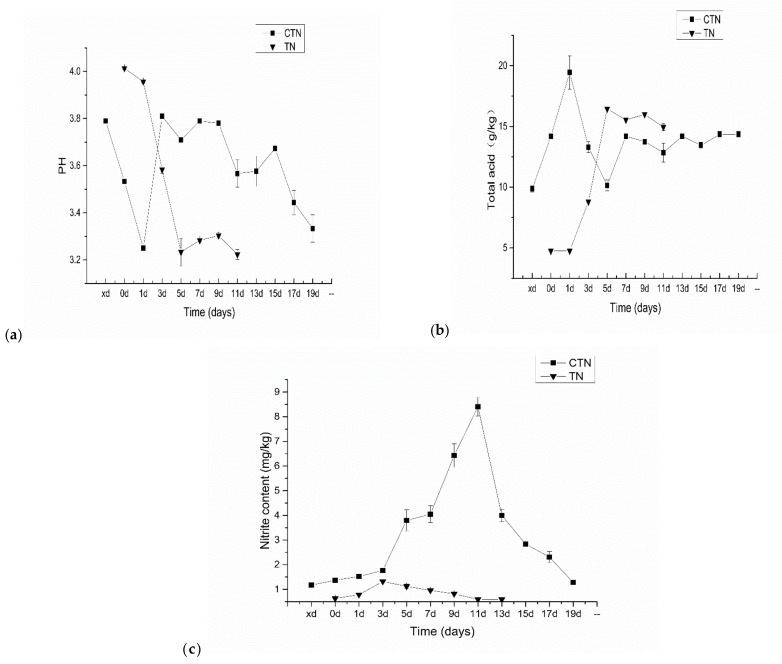
Changes in physicochemical indexes during TN and CTN fermentation: (**a**) pH, (**b**) total acid, (**c**) nitrite content. TN refers to the enhanced fermentation tomato sour soup sample; CTN refers to the traditional tomato sour soup sample.

pH and total acid content are important characteristics of the fermentation process that affect the growth of microorganisms and the accumulation of metabolites, thereby changing the safety and flavor quality of fermented vegetables [35]. It can be seen from Figure 4a that the initial pH value of the two fermentation methods was around 3.9.

In the first stage of fermentation, the pH value dropped rapidly. The sudden increase in pH during the second stage of fermentation in the CTN group on the first day may be due to the sudden accumulation of metabolites by adding the traditional old sour soup as a starter. In the subsequent fermentation process, with the extension of the fermentation time, the amount of nutrients in the tomato sour soup gradually decreased, the microbial metabolites gradually accumulated, and the pH gradually decreased. In the fermentation process of tomato sour soup, the total acid content of tomato sour soup kept increasing with fermentation time (Figure 4b). On the fifth day, the pH of the CTN group reached 3.72 and the total acid content was 10.15 g/kg, which then slowly decreased to a final pH of 3.3, and increased to a final total acid content of 14.35 g/kg. For the TN group, on the fifth day, the total acid content was 16.44 g/kg and the pH was 3.22. TN reached the same final pH as the CTN group on the fifth day, which can be understood as the rapid growth of H1 within 5 days after inoculation and fermentation, leading to an accumulation of lactic acid and a decrease in pH. The change in pH during the fermentation of the TN group is the same as that of traditional Chinese fermented vegetables (3.20–4.50) [36].

One of the most important safety indicators in fermented vegetable products is the nitrite content. As well known, nitrite can cause a variety of harms to the human body, mainly including causing methemoglobinemia and the formation of carcinogenic nitrosamines [37]. A large number of studies have confirmed that the nitrite content in the process of pickling vegetables shows a trend of first increasing and then decreasing, and there is a peak in NO^2−^ or nitrite [38]. It can be seen from Figure 4c that the nitrite peak content reached a maximum on the 11th day of fermentation, which was 8.40 mg/kg, and did not exceed the health and safety standard (20 mg/kg). Yan et al. [39] found that the initial stage of fermentation caused natural fermentation to more easily form a “nitrite peak” because the content of lactic acid bacteria in the initial stage of fermentation is lower, so bacteria containing nitrate reductase are not inhibited and reduce nitrate to nitrite. The nitrite peak in TN appeared on the third day (1.323 mg/kg) and was always close to the minimum concentration of nitrite in CTN. After that, it stabilized. *Lacticaseibacillus* can decompose nitrite and reduce nitrite content in tomato sour soup [18]. Therefore, the inoculation of cultures with pure starters was more effective than spontaneous fermentation in controlling the nitrite concentration of fermented tomato sour soup.

### 3.5. HPLC Detection of Organic Acids

The type and content of organic acids are important factors affecting the flavor quality of sour soup [40]. A total of nine organic acids, including oxalic acid, tartaric acid, malic acid, lactic acid, acetic acid, citric acid, succinic acid, propionic acid, and fumaric acid in CTN and TN samples, were detected by HPLC. It can be seen from Table 2 that lactic acid (28.72 mg·mL^−1^), followed by succinic acid (15.26 mg·mL^−1^), acetic acid (9.18 mg·mL^−1^), and citric acid (3.92 mg·mL^−1^) contribute greatly to CTN fermentation, which is consistent with the results reported by Lin et al. in the literature [12].

The organic acids that contribute greatly to the TN group are lactic acid (31.26 mg·mL^−1^), followed by succinic acid (7.42 mg·mL^−1^), citric acid (12.85 mg·mL^−1^), and acetic acid (6.2 mg·mL^−1^). Lactic acid has a soft, refreshing taste and is the characteristic sour substance of red sour soup. The lactic acid content of the TN group was higher than that of the CTN group, probably because the *Lacticaseibacillus* became the dominant strain to dominate the fermentation process, so that more energy substances were used to produce lactic acids [41]. The concentration of lactic acid in the TN group reached 30.09 mg·ml^−1^ after 7 days of fermentation (D1), which was higher than the 1.73 mg·ml^−1^ in the CTN from the second round of fermentation for 19 days (F) (28.72 mg·mL^−1^), and the lactic acid and succinic acid in the TN group were higher than those in the CTN group after 2 months of fermentation (F1). Succinic acid is a short-chain fatty acid (SCFA). SCFAs refer to organic fatty acids with carbon chains 1–6 [42], which can reduce pH and transmission time in the colon and promote intestinal peristalsis [43]. The content of citric acid in the TN group at the initial stage of fermentation is higher than that in the CTN group. It has been reported that oxalic acid and citric acid have stronger abilities to degrade nitrite than other organic acids [44].

Therefore, by combining the changes in pH, total acid, nitrite content, and organic acid during the fermentation of TN and CTN, it can be concluded that the enhanced fermentation process can be terminated on the seventh day, while the natural fermentation process needs at least 40 days. These results indicate that the fermentation speed was accelerated after the inoculated of strain H1, indicating that, when strain H1 is present, the inoculation fermentation rate is faster. Therefore, using *Lacticaseibacillus casei* H1 as a starter can shorten the production cycle and significantly improve the production efficiency of sour soup.

### 3.6. GC–IMS Analysis for VFCs

To understand the flavor characteristics of tomato sour soup, GC–IMS technology was used to analyze the changes of VFCs during the fermentation of the TN and CTN groups. It can be seen from Table 3 that a total of 43 VFCs were detected in the TN group and a total of 44 VFCs were detected in the CTN group

**Figure 5:**
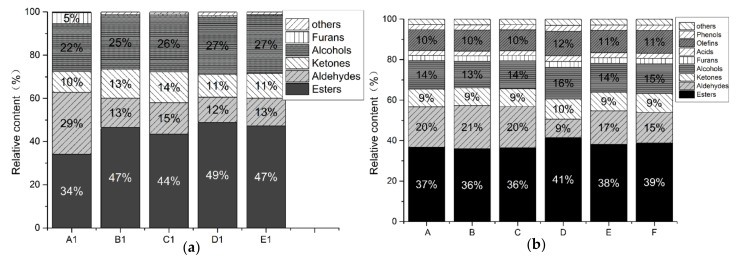
The relative content of VFC during the fermentation of TN (**a**) and CTN (**b**). Notes: TN refers to the enhanced fermentation tomato sour soup sample (A1–E1); CTN refers to the traditional tomato sour soup sample (A–F). Each type of flavor is represented by a unique color. Each column represents a different studied sample.

The TN group includes esters (13 types), alcohols (9 types), aldehydes (12 types), ketones (3 types), olefins (1 type), acids (1 type), furans (2 types), and 2 other types of VFCs; the CTN group includes esters (17 types), alcohols (8 types), aldehydes (8 types), ketones (4 types), olefins (3 types), acids (1 species), phenols (1 species), furans (1 species), and 1 other VFC. It can be seen that esters, alcohols, and aldehydes are the main VFCs in the two groups of samples. The VFCs of the two groups of samples were mostly similar. Figure 5a,b show that the relative contents of esters (49%) and alcohols (27%) in the TN group on day 7 (D) is significantly higher than that of the relative content of esters (39%) and alcohols at the end of CTN fermentation (stage F) (15%).

Ester compounds mostly provide food with fruity, floral, and honey scents, which can strengthen and polish food scents [45]; alcohols give food a rice wine-type scent [46], with the esterification reaction of alcohols and fatty acids being one of the sources of this scent and with the synthesis and metabolism of higher alcohols by microorganisms under the action of acetyltransferase being the other [47]. The above results showed not only that the TN group can achieve similar flavors to the CTN group but also that the content of VFCs in the TN group is generally higher than that in the CTN group, with ethyl acetate and isoamyl acetate being the most prominent. These esters provide a satisfying fruity taste, including banana, apple, pineapple, and pear tastes [48]. These results show the feasibility of improving the flavor quality and rapid fermentation of tomato sour soup with the aim of flavor fidelity.

### 3.7. CTN Multi-Factor Analysis

#### 3.7.1. Correlation Analysis of Microorganisms and Physicochemical Indices during CTN Fermentation

**Figure 6:**
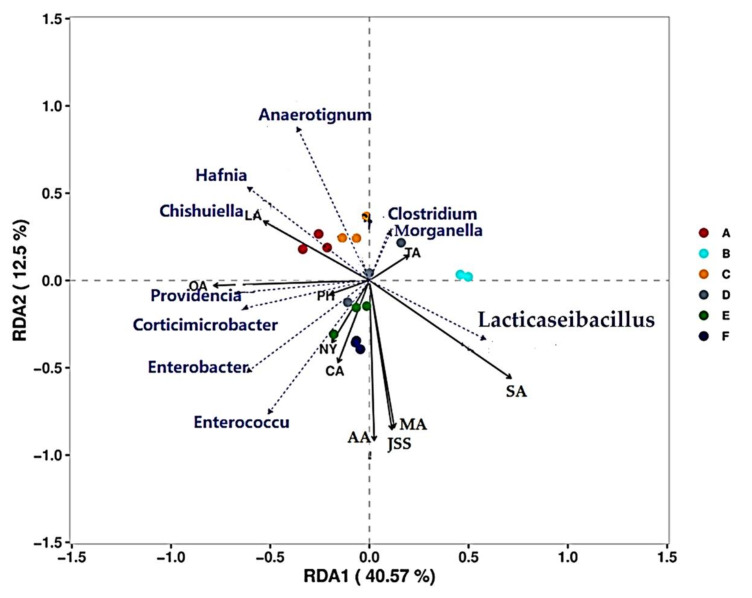
Correlation between RDA (OTU > 0.1%) and physicochemical index for bacterial genera. (A–F) represent samples during traditional tomato sour soup (CTN) fermentation. PH, TA—total acid content; NY—nitrite content; JSS—tartaric acid content; MA—malic acid content; LA—lactic acid content; AA—acetic acid content; CA—citric acid content; SA—succinic acid content; OA—oxalic acid.

**Figure 7:**
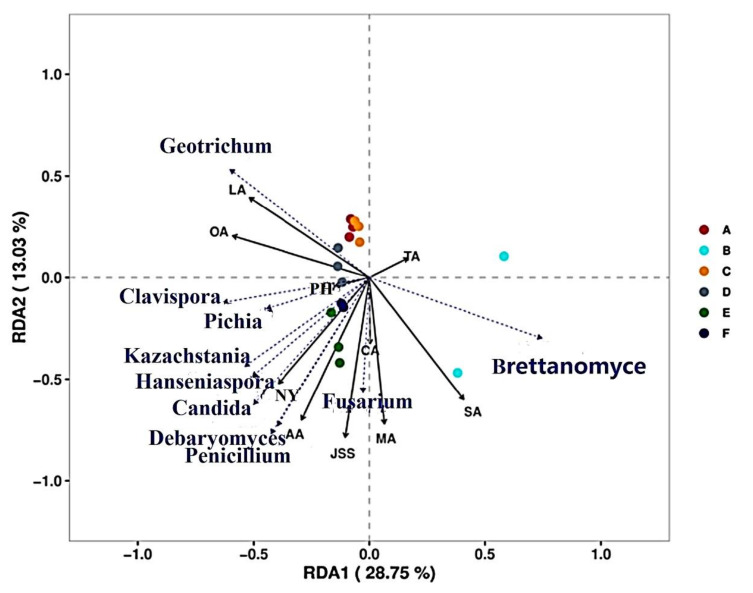
Correlations between RDA (OTU > 0.1%) and physicochemical indices for fungi. (A–F) represent samples during traditional tomato sour soup (CTN) fermentation. PH, TA—total acid content; NY—nitrite content; JSS—tartaric acid content; MA—malic acid content; LA—lactic acid content; AA—acetic acid content; CA—citric acid content; SA—succinic acid content; OA—oxalic acid.

Environmental factors determine changes in the structure of microbial flora. RDA is used to identify the correlation between microbial flora and environmental variables (Figure 6 and Figure 7). An RDA analysis showed that the microbial flora was affected by pH, TA, organic acids, and NY. In terms of bacterial flora (Figure 6), bacterial flora were concentrated in the second and third quadrants and correlated with LA, PH, NY, OA, and CA. *Anaerotignum*, *Hafnia*, and *Chishuiella* were positively correlated with the early stages of fermentation, and *Lacticaseibacillus*, *Providencia*, *Corticimicrobacter*, *Enterobacter*, and *Enterococcus* were correlated with the second stage of fermentation. The dominant bacterium *Lacticaseibacillus* had the highest correlation with the old sour soup (B) in the first year of fermentation. *Lacticaseibacillus*, as the core dominant bacteria during CTN fermentation, showed completely negative correlations with most bacterial genera, especially with *Anaerotignum*, *Hafnia*, and *Chishuiella*, with a strong rejection; however, it was positively correlated with physicochemical indices such as pH, OA, SA, TA, AA, JSS, and MA. Among the fungi (Figure 7), *Pichia* and *Brettanomyce* are the core dominant fungi in the CTN fermentation process. *Pichia* is positively correlated with PH, NY, OA, CA, AA, and JSS, while *Brettanomyce*is is positively correlated with SA, TA, and MA and has a strong repulsive relationship with *Geotrichum*. The results showed that *Lacticaseibacillus* gradually increased during the CTN fermentation process and became the dominant genus, significantly lowering the pH value and increasing the content of organic acids such as OA, SA, AA, JSS, and MA.

#### 3.7.2. Correlation Analysis between Microorganisms and Key VFCs during CTN Fermentation

**Figure 8:**
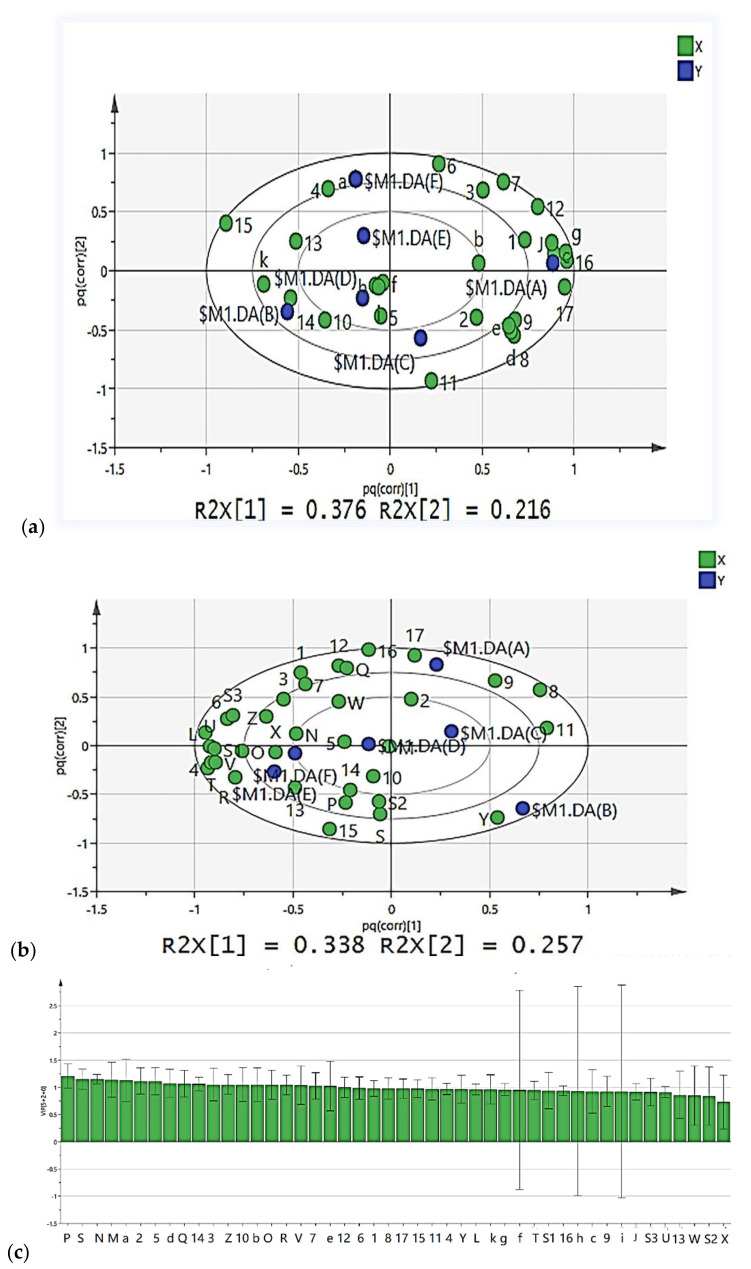
The CTN fermentation process based on the two-way orthogonal partial least squares (O2PLS) method, with diagrams (**a**): the bacterial genus level (VIP > 1, diagram c) in relation to VFCs (VIP > 1, diagram c); diagram (**b**): the fungal genus level (VIP > 1, diagram c) in relation to VFCs (VIP > 1, diagram c); diagram (**c**); the bacterialgenus level (VIP > 1), the fungal genus level (VIP > 1) and VFCs (VIP > 1).CTN refers to the traditional tomato sour soup sample; 1—2-pentylfuran, 2—alpha-Terpinene, 3—methional, 4—2-butanone, 5—2-octanol, 6—3-pentanone, 7—pentyl acetate, 8—Ethyl Acetate, 9—limonene, 10—ethyl 2-hydroxypropanoate, 11—2-methylpropyl acetate, 12—benzaldehyde, 13—linalool, 14—butanal, 15—6-methyl-5-hepten-2-one, 16—ethyl 2-methylpropanoate, 17—alpha-Pinene; a*—Psychrobacter*, b*—Pseudomonas*, c*—Dysgonomonas*, d*—uncultured_bacterium_o_chloroplast*, e*—Anaerotignum*, f*—Lactococcus*, g*—Chishuiella*, h*—Morganella*, i*—Clostridium*, J*—Paenirhodobacter*, k*—Lacticaseibacillus*; Z*—Pichia*, L*—Kazachstania*, M*—Wickerhamiella*, N*—Trichosporon*, O*—Yamadazyma*, P*—Eremothecium*, Q*—Geotrichum*, R*—Kodamaea*, S*—Malassezia*, T*—Debaryomyces*, U*—Hanseniaspora*, V*—Penicillium*, W*—Kurtzmaniella*, X*—Meyerozyma*, Y*—Brettanomyces*, S1*—Candida*, S2*—Ceratobasidium*, S3*—Clavispora*.

The application of the SIMCA-14.1 software for O2PLS-DA analysis revealed the relationship between bacterial flora, fungal flora, and VFCs in the CTN fermentation process. According to the variable importance for projection (VIP) calculated in the O2PLS-DA analysis model, the contribution of each variable to the classification was quantified [49], and we constructed the O2PLS-DA model (Figure 8a,b). In the model of Figure 8a, R^2^ X = 0.984, R^2^ Y = 0.958, and Q^2^ = 0.878, and in the model of Figure 8b, R^2^ X = 0.935, R^2^ Y = 0.992, and Q^2^ = 0.96, indicating that the O2PLS-DA model is suitable for use in analysis and prediction [50]. The VIP (pred) vector of the analyzed volatile flavor (the VIP value of the predicted component) ranges from 0.821 to 1.418 (Figure 8c), including 17 flavor substances (VIP (pred) > 1, with the VIP (pred) vector (VIP value of the predicted component) ranging from 0.70 to 1.558); 29 microbial genera (VIP (pred) > 1), including 11 species of bacteria (VIP(pred): 1–1.558); and 18 species of fungi (VIP(pred): 1.011–1.194). These bacteria and fungi may play important roles in the biosynthesis of volatile components.

At the level of the 11 bacterial genera (VIP(pred) > 1) (8-c), *Psychrobacter* (a) and 2-butanone (4), linalool (13), 6-methyl-5-hepten-2-one (15), and other volatile flavor substances are positively correlated; *Lactococcus* (f), *Morganella* (h), *Clostridium* (i), *Lacticaseibacillus* (k), and 2-octanol (5), ethyl 2-hydroxypropanoate (10), butanal (14), and other flavor substances are positively correlated; as the most dominant genus in CTN, *Lacticaseibacillus* (k) is significantly positively correlated with 11 VFCs and negatively correlated with many volatile components (2-pentylfuran (1), methional (3), 3-pentanone (6), pentyl acetate (7), benzaldehyde (12), and ethyl 2-methylpropanoate (16)), indicating that *Lacticaseibacillus* plays an important role in the formation of VFCs in CTN. We also found that the dominant genus *Lacticaseibacillus* (k) was negatively correlated with *Pseudomonas* (b), J-*Paenirhodobacter*, c-*Dysgonomonas*, probably because the dominant *Lacticaseibacillus* (k) can convert carbohydrates into large amounts of acetic acid through heterofermentation, thus acidifying the environment and eventually inhibiting the growth of other microorganisms. The dominant *Lacticaseibacillus* (k) has a strong positive correlation with the B-stage samples, which is in line with the characteristics of lactic acid fermentation.

At the level (8-c) of the 18 fungal genera (VIP > 1), *Pichia* (Z) and *Geotrichum* (Q) *Brettanomyces* (Y) are considered the core fungal genera of volatile components. *Pichia* (Z), *Geotrichum* (Q) and 2-pentylfuran (1), methional (3), 2-octanol (5), 3-pentanone (6), pentyl acetate (7), benzaldehyde (12), ethyl 2-hydroxypropanoate(10), and seven kinds of VFCs, including methylpropanoate (16), are positively correlated. *Brettanomyces* (Y) is positively correlated with four types of VFCs: ethyl 2-hydroxypropanoate (10), linalool (13), butanal (14), and 6-methyl-5-hepten-2-one (15), indicating that the core fungus is *Pichia* (Z) and that *Brettanomyces* (Y) plays a vital role in the formation of VFCs in CTN. Additionally, some studies have shown that yeast plays an important role in the formation of VFCs during the fermentation process.

## 4. Conclusions

In this study, the microbial flora and VFCs found during the CTN fermentation process were investigated by using HTS and GC–IMS. Bacterial genera such as *Lacticaseibacillus*, *Enterobacter*, and *Providencia* and fungal genera such as *Pichia*, *Geotrichum*, and *Brettanomyces* were detected in high abundance in different fermentation processes. Moreover, *Lacticaseibacillus* continues to grow, and dominates in the later stages of fermentation. A total of 44 VFCs were detected in CTN, and 11 bacterial and 18 fungal genera were identified as the functional core flora for flavor formation. Additionally, as the most dominant genus in CTN, *Lacticaseibacillus* was significantly positively correlated with 11 VFCs, further validating the importance of this dominant bacteria for VFC production. Then, enhanced fermentation with the *Lacticaseibacillus casei* H1 strain proved that not only was the fermentation period shortened, but the production of nitrite was also reduced. The GC–IMS analysis showed that the contents of esters and alcohols in the TN group were 1.26 times and 1.8 times higher than those in CTN group, respectively. These results provide support for the screening of a tomato sour soup starter and for the industrial production of vegetable fermentation products. This study also found that yeast plays an important role in the development of tomato sour soup flavor. Therefore, the flavor formation mechanism of tomato sour soup fermented by dominant lactic acid bacteria and yeast needs to be further investigated.

## Figures and Tables

**Table 1:** Number of sequences analyzed, observed diversity richness (observed OTUs), community richness index (Chao 1 and ACE), community diversity index (Shannon and Simpson), and estimated sample coverage for 16S rDNA and ITS rDNA libraries of CTN samples.

Sample	Richness Index				Diversity Index				Coverage	
	ACE		Chao 1		Simpson		Shannon		Bacteria	Fungi
	Bacteria	Fungi	Bacteria	Fungi	Bacteria	Fungi	Bacteria	Fungi		
A	96.41 ± 3.93 ^b^	56.72 ± 9.07 ^a^	94.87 ± 3.45 ^b^	52.19 ± 6.54 ^a^	0.9 ± 0.003 ^a^	0.70 ± 0.02 ^d^	4.09 ± 0.05 ^ab^	2.45 ± 009 ^c^	0.999	0.9992
B	29.87 ± 1.97 ^c^	60.55 ± 14.77 ^a^	27.32 ± 1.36 ^c^	59.50 ± 13.45 ^a^	0.45 ± 0.01 ^b^	0.74 ± 0.01 ^c^	1.55 ± 0.03 ^c^	2.47 ± 0.03 ^c^	0.9997	0.9994
C	106.3 ± 4.7 ^ab^	71.69 ± 10.81 ^a^	105.51 ± 5.20 ^ab^	63.95 ± 6.65 ^a^	0.89 ± 0.01 ^a^	0.67 ± 0.03 ^e^	4.00 ± 0.08 ^b^	2.43 ± 0.15 ^c^	0.9987	0.999
D	121.17 ± 26.52 ^a^	64.22 ± 12.29 ^a^	122.89 ± 15.01 ^a^	66.86 ± 12.92 ^a^	0.91 ± 0.01 ^a^	0.77 ± 0.01 ^b^	4.19 ± 0.19 ^a^	2.89 ± 0.05 ^b^	0.9983	0.9991
E	103.8 ± 8.53 ^ab^	73.198 ± 15.50 ^a^	114.32 ± 19.22 ^ab^	72.53 ± 18.22 ^a^	0.90 ± 00.003 ^a^	0.79 ± 0.01 ^ab^	4.09 ± 0.05 ^ab^	3.13 ± 0.08 ^a^	0.9988	0.9991
F	100.64 ± 4.47 ^ab^	66.52 ± 6.88 ^a^	100.56 ± 5.39 ^b^	64.86 ± 7.67 ^a^	0.90 ± 0.002 ^a^	0.82 ± 0.01 ^a^	4.11 ± 0.02 ^ab^	3.17 ± 0.06 ^a^	0.999	0.9992

Values are expressed as the mean ± standard error (*n* = 3), ^a–e^ different letters in the same column indicate significant differences (*p* < 0.05). (A–F) represent samples during traditional tomato sour soup (CTN) fermentation.

**Table 2:** Content of organic acids (mg·mL^−1^) during the fermentation of CTN and TN.

Category	Oxalic Acid	Tartaric Acid	Malic Acid	Lactic Acid	Acetic Acid	Citric Acid	Succinic Acid	Propionic Acid	Fumaric Acid
A (mg·mL^−1^)	0.37 ± 0.05 ^d^	0.84 ± 0.01 ^cd^	1.22 ± 0.05 ^f^	27.09 ± 0.06 ^ef^	5.93 ± 0.06 ^d^	4.33 ± 0.11 ^f^	9.03 ± 0.05 ^g^	0.2 ± 0.12 ^f^	0
B (mg·mL^−1^)	0.15 ± 0.06 ^f^	1.02 ± 0.03 ^bc^	2.49 ± 0.01 ^d^	17.22 ± 0.04 ^h^	6.71 ± 0.05 ^cd^	3.45 ± 0.06 ^h^	16.23 ± 0.17 ^b^	1.3 ± 0.05 ^b^	0
C (mg·mL^−1^)	0.18 ± 0.05 ^ef^	0.63 ± 0.04 ^de^	0.53 ± 0.05 ^h^	44.28 ± 0.51 ^a^	6.09 ± 0.05 ^d^	1.88 ± 0.05 ^j^	12.93 ± 0.05 ^f^	0.67 ± 0.12 ^e^	0
D (mg·mL^−1^)	0.22 ± 0.05 ^e^	1.04 ± 0.02 ^bc^	0.36 ± 0.02 ^i^	25.08 ± 0.12 ^g^	7.37 ± 0.02 ^c^	2.74 ± 0.05 ^i^	14.29 ± 0.11 ^e^	0.93 ± 0.06 ^d^	0
E (mg·mL^−1^)	0.23 ± 0.04 ^e^	1.33 ± 0.03 ^a^	3.66 ± 0.02 ^c^	27.18 ± 0.12 ^ef^	8.31 ± 0.57 ^b^	3.82 ± 0.12 ^g^	14.52 ± 0.17 ^d^	1.06 ± 0.06 ^c^	0
F (mg·mL^−1^)	0.24 ± 0.01 ^e^	1.21 ± 0.05 ^ab^	4.1 ± 0.06 ^b^	28.72 ± 0.12 ^d^	9.18 ± 0.58 ^a^	3.92 ± 0.05 ^g^	15.26 ± 0.12 ^c^	1.14 ± 0.017 ^c^	-
A1 (mg·mL^−1^)	0.37 ± 0.01 ^d^	0.71 ± 0.52 ^de^	4.85 ± 0.10 ^a^	3.12 ± 0.06 ^j^	3.62 ± 0.05 ^e^	7.5 ± 0.02 ^e^	0.85 ± 0.01 ^l^	0	0.71 ± 0.01 ^b^
B1 (mg·mL^−1^)	1.22 ± 0.01 ^b^	0.47 ± 0.01 ^e^	1.56 ± 0.03 ^e^	5.5 ± 0.05 ^i^	1.3 ± 0.58 ^f^	10.38 ± 0.23 ^d^	2.25 ± 0.02 ^k^	0	0.33 ± 0.03 ^c^
C1 (mg·mL^−1^)	1.4 ± 0.01 ^a^	0.41 ± 0.01 ^e^	1.57 ± 0.09 ^e^	26.57 ± 0.58 ^f^	3.88 ± 0.01 ^e^	11.98 ± 0.58 ^c^	3.54 ± 0.14 ^j^	0	0.02 ± 0.01 ^d^
D1 (mg·mL^−1^)	0.38 ± 0.06 ^d^	0.49 ± 0.03 ^e^	0.95 ± 0.03 ^g^	30.09 ± 0.57 ^c^	6.66 ± 0.58 ^cd^	12.51 ± 0.05 ^b^	6.68 ± 0.05 ^i^	0	0.03 ± 0.01 ^d^
E1 (mg·mL^−1^)	0.78 ± 0.01 ^c^	0.49 ± 0.02 ^e^	0.45 ± 0.06 ^hi^	31.26 ± 0.58 ^b^	6.2 ± 0.57 ^d^	12.85 ± 0.05 ^a^	7.42 ± 0.05 ^h^	0	0.02 ± 0.01 ^d^
F1 (mg·mL^−1^)	0.23 ± 0.01 ^e^	0.62 ± 0.06 ^de^	1.2 ± 0.08 ^f^	30.75 ± 0.57 ^e^	6.52 ± 0.57 ^d^	7.28 ± 0.17 ^e^	18.54 ± 0.12 ^a^	1.44 ± 0.05 ^a^	1.95 ± 0.01 ^a^

Values are expressed as the mean ± standard error (*n* = 3); different letters (a–l) in the same column indicate significant differences (*p* < 0.05). (A–F) represent samples during traditional tomato sour soup (CTN) fermentation. (A1–F1) represent the samples during the fermentation process of the enhanced fermentation tomato sour soup (TN).

**Table 3:** Comparison of volatile compounds.

Compound	Retention Index	Retention Time/s	Migration Time/ms	F	D1
Esters					
Methyl Salicylate	1235.7	688.365	1.20588	424.79	–
Butyl butanoate	1004.3	356.079	1.81327	280.95	–
Pentyl acetate	912.8	274.089	1.76114	239.64	154.88
Isoamyl acetate	875	247.755	1.75345	224.98	6023.73
Ethyl 2-methylbutanoate	848.8	234.134	1.66369	216.25	–
Ethyl 2-hydroxypropanoate	818.5	218.374	1.54705	206.15	–
Ethyl butanoate	792.4	204.821	1.56923	197.52	105.65
Butyl acetate	806.1	211.905	1.63724	202.08	–
Methyl 2-methylbutanoate	769.8	194.685	1.54033	190.52	–
2-Methylpropyl acetate	764	192.337	1.62702	188.86	–
Ethyl 2-methylpropanoate	748.9	186.175	1.58294	184.37	268.66
Ethyl propanoate	705.9	168.646	1.46174	171.59	–
Ethyl Acetate	596.7	137.37	1.34657	143.45	13898.89
Isopropyl acetate	654.9	152.715	1.48967	158.07	–
Propyl butanoate	898.9	262.349	1.69733	233.57	–
Methyl hexanoate	923.8	283.382	1.6923	244.39	–
Ethyl heptanoate	1100.3	493.969	1.92705	340.83	–
Ethyl hexanoate-M	1005.5	357.86	1.33	–	334.68
Ethyl hexanoate-D	1006.3	359.03	1.81	–	1157.28
Methyl hexanoate	923.9	283.5	1.69	–	265.26
Ethyl butanoate	793.3	205.29	1.57	–	5383.76
2-Methylpropyl acetate	768	193.94	1.63	–	1753.84
Propyl acetate	706.3	168.82	1.47	–	1665.82
Methyl acetate	552.8	125.76	1.19	–	1256.93
Hexyl acetate	1016.4	373.57	1.91	–	591.28
Olefins					
Limonene	1031.8	395.617	1.21701	297.79	307.08
alpha-Terpinene	1025.4	386.493	1.22695	293.83	–
alpha-Pinene	946.6	302.684	1.22113	254.07	–
Aldehydes					
2-Decenal	1267	733.342	1.47569	444.44	–
(E)-2-Heptenal	959.3	313.499	1.27664	259.65	–
Benzaldehyde	961.5	315.324	1.47823	260.7	–
3-Methylbutanal	653.1	152.236	1.41542	157.58	308.79
2-Methylbutanal	679.4	159.186	1.39664	164.15	–
Butanal	579.2	132.737	1.304	139.05	–
(E)-2-Hexenal	864.7	242.411	1.52651	221.46	2919.78
Vanillin	1398.9	922.715	1.26926	526.49	–
Nonanal	1110.2	508.25	1.48	–	115.96
(E)-2-Octenal-M	1058.6	434.04	1.34	–	488.64
(E)-2-Octenal-D	1058.6	434.04	1.83	–	117.27
(E)-2-Heptenal-D	955	309.85	1.68	–	58.54
(Z)-2-Hexenal	844.9	232.09	1.53	–	887.34
Furfural	828.5	223.58	1.34	–	399.64
(E)-2-Pentenal	749.4	186.37	1.37	–	244.06
Propanal	523.5	118.04	1.15	–	1403.93
Pentanal	702.3	167.22	1.41	–	895.79
Ketones					
6-Methyl-5-hepten-2-one	993.1	342.088	1.18294	274.31	4247.15
3-Pentanone	691.6	162.854	1.34156	167.31	438.19
2-Pentanone	695.4	164.38	1.37	–	2387.85
2-Butanone	572.5	130.984	1.25643	137.36	
Alcohols					
2-Octanol	1004.7	356.687	1.45694	281.03	–
(E)-3-hexen-1-ol	835.7	227.325	1.54284	211.85	–
3-Methylpentanol	840.2	229.658	1.60397	213.37	–
2-Hexanol	803.7	210.673	1.58697	201.26	1985.79
3-Methyl-1-butanol	727.7	177.527	1.47801	178.04	–
Propanol	543	123.195	1.27084	129.99	487.59
2-Propanol	510.4	114.57	1.20354	121.8	4498.07
Linalool	1104.7	500.263	1.2256	343.2	
Ethanol	466.1	102.865	1.14036	110.69	8048.69
1-Hexanol	875.8	248.17	1.64	–	1985.79
2-Methyl-1-butanol	751.7	187.32	1.49	–	113.88
2-Pentenal	738.2	181.83	1.36	–	381.44
Isopentanol	728.6	177.9	1.5	–	1914.83
Isohexanol	845.2	232.27	1.62	–	316.96
1-Pentanol	761	191.09	1.51	–	71.46
Acids					
Hexanoic acid	938.5	295.882	1.66882	250.8	
Propanoic acid	714.4	172.15	1.26	–	92.86
Phenols					
Methional	986	336.005	1.48249	271.35	–
Furans					
2-Ethylfuran	682.9	160.13	1.32	–	356.27
2-Pentylfuran	997.1	345.738	1.2596	276.18	90.3
others					
2-Ethyl pyrazine	924.6	284.1	1.52	–	317.48
2-Acetylthiazole	1015.3	371.894	1.49811	287.65	821.19

Note: From left to right in the table, F and D1 are the peak volumes of flavor component ions detected by the two samples: F:CTN group fermented for 19 days; D1:TN fermentation for 7 days.

## Data Availability

Not applicable.
